# Molecular and Clinical Features of Adrenocortical Tumors in Beckwith–Wiedemann Spectrum

**DOI:** 10.3390/cancers16233967

**Published:** 2024-11-26

**Authors:** Diana Carli, Federico Rondot, Maria Luca, Anna Campello, Stefano Gabriele Vallero, Elisa Tirtei, Andrea Gazzin, Simona Cardaropoli, Francesca Montanari, Claudio Graziano, Paola Quarello, Abu Saadat, Angela Sparago, Giovanni Battista Ferrero, Franca Fagioli, Alessandro Mussa

**Affiliations:** 1 Department of Medical Sciences, University of Torino, 10126 Torino, Italy; diana.carli@unito.it (D.C.); federico.rondot@unito.it (F.R.); maria.luca@unito.it (M.L.); 2 Immunogenetics and Transplant Biology Unit, Città della Salute e della Scienza University Hospital, 10126 Torino, Italy; 3 Pediatric Onco-Hematology, Regina Margherita Children’s Hospital, Città della Salute e della Scienza University Hospital, 10126 Torino, Italy; acampello@cittadellasalute.to.it (A.C.); svallero@cittadellasalute.to.it (S.G.V.); elisa.tirtei@unito.it (E.T.); paola.quarello@unito.it (P.Q.); franca.fagioli@unito.it (F.F.); 4 Department of Public Health and Pediatrics, University of Torino, 10126 Torino, Italy; andrea.gazzin@unito.it (A.G.); simona.cardaropoli@unito.it (S.C.); 5 Medical Genetics Unit, IRCCS Azienda Ospedaliero-Universitaria di Bologna, 40138 Bologna, Italy; francesca.montanari@aosp.bo.it; 6 Medical Genetics Unit, AUSL della Romagna, 48121 Ravenna, Italy; claudio.graziano2@auslromagna.it; 7 Department of Environmental Biological and Pharmaceutical Sciences and Technologies (DiSTABiF), Università degli Studi della Campania “Luigi Vanvitelli”, 81100 Caserta, Italy; abu.saadat@unicampania.it (A.S.); angela.sparago@unicampania.it (A.S.); 8 Department of Clinical and Biological Sciences, University of Torino, 10043 Orbassano, Italy; giovannibattista.ferrero@unito.it; 9 Clinical Genetics Unit, Regina Margherita Children’s Hospital, Città della Salute e della Scienza University Hospital, 10126 Torino, Italy

**Keywords:** Beckwith–Wiedemann spectrum, adrenocortical tumors, adrenocortical carcinoma

## Abstract

Background/Objectives: Adrenocortical tumors (ACTs), including adrenocortical adenoma (ACA) and carcinoma (ACC), represent 0.3–0.4% of pediatric tumors. Beckwith–Wiedemann spectrum (BWSp) confer an increased risk of ACTs, but prognosis, management, and associated molecular characteristics are unclear. Methods: This paper combines a literature review of 54 published cases of BWSp-ACT with a report of one newly identified patient, totaling 55 cases with a confirmed BWSp clinical and/or molecular diagnosis. Results: Nineteen patients with ACA, 33 with ACC, and 3 with ACT of uncertain malignant potential (umACT) were included. Twenty patients had uniparental disomy of chromosome 11p15.5 (patUPD11), 11imprinting Center 2 Loss-of-methylation (IC2-LoM), and had 2 11p15 locus duplication. Eleven patients were diagnosed during cancer screening procedures, including two metastatic at diagnosis ACC. Conclusions: Almost half of ACC patients reached the minimum score for clinical BWSp diagnosis only after ACC onset, suggesting that the BWSp score has limited value for the early diagnosis in such a setting. Two patients with metastatic ACC had a histopathological Wieneke score ≤2, not correlating with clinical malignancy and confirming limitations of the current histopathological classification, as previously documented. Ultrasound screening failed identifying the ACC before metastasis in two cases, indicating an urgent need to develop new strategies for screening of ACTs in BWSp. Furthermore, some cases of metastatic ACC exhibited unexpectedly indolent behavior despite being malignant.

## Simple Summary

Adrenocortical tumors (ACTs) are rare in children and can be benign (adrenocortical adenoma or ACA) or malignant (adrenocortical carcinoma or ACC). Children with Beckwith–Wiedemann spectrum (BWSp) are at an increased risk of developing ACTs, but there is uncertainty around the prognosis, management, and molecular characteristics associated with these tumors. In this study, we provide a literature review of data from 54 published patients with BWSp-ACT and report on one additional new patient, totaling 55 cases: 19 ACA, 33 ACC, and 3 uncertain malignant potential (umACT). Almost half of the ACC patients were clinically diagnosed with BWSp after ACC onset, suggesting that the BWSp clinical diagnostic score has limited value for early diagnosis in this setting. Two patients with metastatic ACC had a low histopathological Wieneke score, confirming limitations of the current histopathological classification, as previously documented. Ultrasound screening failed to identify ACC before metastasis in two cases, indicating a need for new screening strategies for ACTs in children with BWSp. Notably, in some cases, metastatic ACC exhibited unexpectedly indolent behavior, despite their malignancy.

## 1. Introduction

Beckwith–Wiedemann Spectrum (BWSp) is an overgrowth condition caused by genetic or epigenetic alterations of the 11p15.5 chromosome region. A molecular diagnosis can be found in approximately 80% of patients [1]. Clinical diagnosis is based on the identification of cardinal features such as macroglossia, omphalocele, lateralized overgrowth, and multifocal and/or bilateral Wilms tumor (WT). In addition to these primary features, BWSp is associated with an increased risk of embryonal tumors in early childhood, such as hepatoblastoma, neuroblastoma, rhabdomyosarcoma, and adrenocortical carcinoma (ACC), which significantly impact patient outcomes [1]. Early-onset ACC has an estimated incidence <1% in individuals with BWSp [2]. In children with BWSp, cancer screening with abdominal ultrasound (USS) is recommended to facilitate early detection and management of WT [3], the most frequent tumor in BWSp; however, USS can also detect hepatoblastomas and adrenocortical tumors (ACTs), although there are limited data on the utility of such screening strategies for those tumors [1,4].

ACTs are rare, with an incidence between 0.7 and 2 cases per million people annually [2]. ACC, a malignant form of these tumors, is more common in adults, typically presenting between the ages of 40 and 50. However, pediatric ACTs are exceedingly rare, usually occurring before the age of 5 [2]. In children, most ACTs secrete hormones and present with endocrine abnormalities, in contrast to adults, where non-functional tumors are more common [2]. Pediatric ACTs, including ACC, can be influenced by genetic syndromes such as Li–Fraumeni syndrome and BWSp, emphasizing the importance of genetic risk factor identification [2]. The prognosis and treatment strategies for ACTs depend on the tumor type, stage, and individual patient characteristics [2].

The histopathological classification of ACTs is crucial for patient management, as it aids in determining prognosis and appropriate treatment. The modified Weiss system, developed in 2004, includes features such as nuclear grade and invasion to enhance prognostic accuracy [5]. A more widely accepted system is the Wieneke score, proposed by Wieneke and colleagues in 2003, which assesses tumor architecture and factors like necrosis, hemorrhage, and atypical mitoses [5]. However, recent studies suggest that these scoring systems are less reliable in predicting prognosis for pediatric tumors, in particular low-stage ones, making clinical decision making complex [5,6].

Treatment of pediatric ACC typically involves surgical resection, with the goal of complete removal of the tumor. Depending on the stage and risk of recurrence, adjuvant therapies such as chemotherapy, mitotane, and, in some cases, radiation therapy may be employed [2]. Despite aggressive treatment, the overall prognosis for pediatric ACC is poor, with a 5-year survival rate of 30–40%. However, children with low-stage disease who achieve complete surgical resection have better outcomes [2]. Ongoing research is exploring novel treatment strategies, including targeted therapies and immunotherapy, which offer hope for improved survival rates in pediatric ACC [2].

In the present study, we reviewed data from 1 newly reported and 54 previously published patients with BWSp-associated ACT, including those with a confirmed clinical and/or molecular BWSp diagnosis, to better understand the clinical characteristics and outcomes of this rare but significant BWSp complication.

## 2. Materials and Methods

Newly reported cases were identified through a patient call coordinated by the Italian Association for Patients with BWSp (AIBWS.org) within the network of Italian hospitals treating BWSp.

Pyrosequencing analysis and SNP array analysis were carried out as previously reported [7,8].

Published cases were retrieved through a comprehensive literature review, with a focus on BWSp patients with ACT and/or ACC. The search strategy targeted articles that specifically mentioned ‘Beckwith-Wiedemann spectrum’, ‘Beckwith-Wiedemann syndrome’, and ‘Beckwith Wiedemann’, as well as the key BWSp clinical features, such as ‘macroglossia’, ‘hemihypertrophy’, ‘lateralized overgrowth’, and ‘omphalocele’. Additionally, terms related to adrenocortical tumors, including ‘adrenocortical adenoma’, ‘adrenocortical carcinoma’, ‘ACTs’, ‘ACA’, and ‘ACC’, were employed to ensure the inclusion of all relevant studies. When the Wieneke histopathological score [5] or the BWSp clinical diagnostic score [1] were not explicitly provided in the articles, we calculated these scores, when feasible, based on the data reported in the respective studies.

Statistical analysis to compare clinical and molecular features between patients with ACA or uncertain malignant potential ACT (umACT) and those with ACC was performed. For categorical variables, including overall mortality, molecular diagnosis, clinical presentation, and BWSp features, Fisher’s exact test was used due to small sample sizes and expected frequencies. Continuous variables, such as age at diagnosis and BWSp clinical score, were analyzed using the Mann–Whitney *U* test to account for non-normal distributions. A *p*-value of less than 0.05 was considered statistically significant.

## 3. Results

### 3.1. Case Report

We report two cases. The first case involves a female newborn hospitalized for a retroperitoneal mass associated with BWSp features, including macrosomia, macroglossia, lateralized overgrowth, umbilical hernia, ear lobe anomalies, and neonatal hypoglycemia. The mass was surgically removed, and histopathological analysis confirmed an adrenal cortical neoplasm with neuroendocrine dedifferentiation. TNM staging classified the tumor as pT3, pNx, pMx, or R1, with a high-risk category based on stage, mitotic index, and score. Methylation-specific MLPA analysis of chromosome 11p15.5 on peripheral blood, confirmed by multiple samples, showed mild hypermethylation of Imprinting Center 1 (IC1-GoM) and mild Loss-of-methylation at Imprinting Center 2 (IC2-LoM), suggestive of mosaic segmental paternal uniparental disomy of chromosome 11 (UPD(11)pat), although SNP-array analysis did not confirm UPD(11)pat.

**Figure 1:**
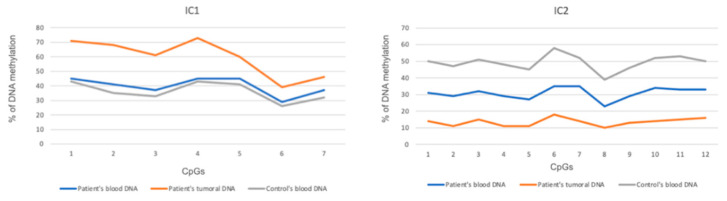
DNA methylation analysis of IC1 and IC2 regions in blood and tumoral DNA samples as measured by bisulfite pyrosequencing. Control is a blood DNA sample from a normal individual.

The second case is an update on a patient initially reported by Pilloni in 2023 [9]. Diagnosed with BWSp due to IC2-LoM at birth, the patient presented a cystic adrenal mass, which progressed to a virilizing solid mass (Wieneke score 2) by age 2 years and 11 months, surgically removed. During staging, brain MRI revealed multiple ACC metastases. He was treated with chemotherapy and mitotane and remains clinically stable at age 6, with slight reduction of the brain lesions. Genetic analysis on brain metastasis samples revealed somatic pathogenic variants in *CTNNB1* (NM_001904.4):c.133T>C, p.Ser45Pro (S45P), and *GNAS* (NM_000516.7):c.602G>A, p.Arg201His (R201H). DNA methylation analysis by bisulfite treatment followed by pyrosequencing confirmed IC2-LoM in the blood and showed more pronounced IC2 hypomethylation with additional IC1-GoM in the primary tumor. SNP-array on primary tumor DNA ruled out UPD(11)pat (Figure 1). Further details of both patients are provided in Table 1.

### 3.2. Literature Review

We retrieved 49 articles spanning from 1952 to 2023 for a total of 54 BWSp patients, 19 with ACA, 32 with ACC, and 3 with uncertain malignant potential (umACT). Patients’ data are detailed in Table 1 (ACC) and Table 2 (ACA and umACT).

### 3.3. ACA and umACT

Nineteen BWSp patients were diagnosed with ACA, of whom only eight underwent molecular characterization: five had IC2-LoM, two had UPD(11)pat, and one carried a duplication of chromosome 11p15.5. The clinical score was calculated for 14 patients, with a mean score of 5.0 ± 2.2. The mean age at diagnosis was 9 years (range 45 days to 45 years, with a median age of 2 years). Only three cases were diagnosed after the age of 18. In 12 out of 19 cases (63%), the ACA diagnosis was based on clinical presentation, primarily virilization and Cushing syndrome, while the remaining cases were identified through ultrasound screening. Thirteen patients had unilateral tumors, while three presented with bilateral asynchronous tumors—one developing after 5 months and two after more than 5 years. Additionally, one patient had an ectopic spinal adenoma (Table 2). Eighteen patients underwent surgery, with follow-up data available for eleven of them. Two patients died due to complications unrelated to the progression of adrenal disease: one from metastatic WT [10] and the other from post-operative sepsis [11]. One patient experienced a local recurrence of a spinal ectopic adenoma [12] nine months after surgery. 

Three additional cases with uncertain malignant potential (umACT) were identified, two of whom carried UPD(11)pat. All three underwent surgical removal, and follow-up data for one patient indicated no complications after 12 years [13].

Six patients underwent molecular testing on tumor DNA: two carried marked IC2-LoM [14,15], two exhibited loss of heterozygosity (LOH) at chromosome 11p15 [16,17], one carried somatic pathogenic variants of *CTNNB1* and *GNAS* genes [13], and one carried somatic pathogenic variants of the *MKRN3* and *CYP17A1* genes [13].

### 3.4. ACC

**Figure 2:**
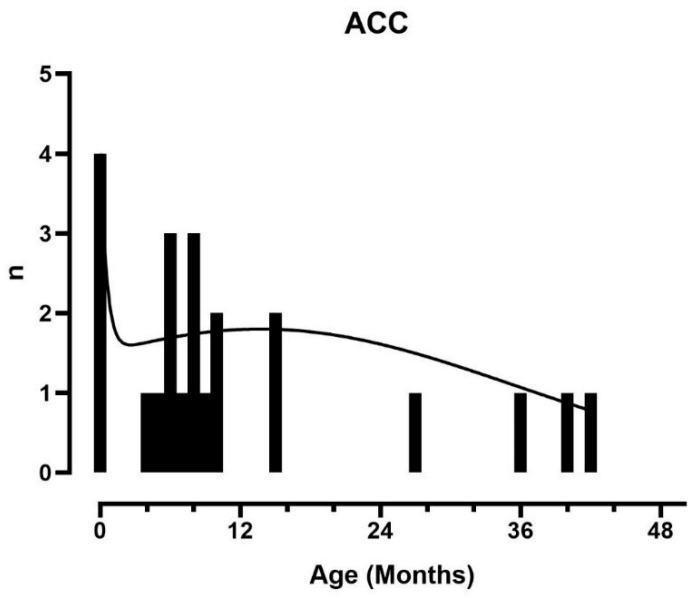
Distribution of age of diagnosis for ACC in BWSp patients.

A diagnosis of ACC in published BWSp patients was identified in 32 cases (1 of which also had a synchronous diagnosis of ACA) and in the newly reported case (see Section 3.1). Molecular characterization was performed on 24 patients: 16 carried UPD(11)pat, 2 of whom were genome-wide UPDpat, 6 patients carried IC2-LoM, and 1 carried a chromosome 11p15.5 duplication. The patients did not undergo additional germline molecular testing beyond that for BWSp; in particular, TP53 germline variants were not excluded, except for the patient described by Pilloni in 2023 [9]. The clinical score was calculated for 26 patients, with a mean score of 3.9 ± 2.1, and 18 patients with a score ≤ 4. The mean age at diagnosis was 4 years (range from zero to 37 years, with a median age of <1 year) (Figure 2). In total, 58% of cases (18/31) were diagnosed before the age of 2 and 80% (25/31) were diagnosed before the age of 5. Only one case was diagnosed after the age of 18. The age at diagnosis in months is summarized in Figure 2. In 22 out of 33 cases (66%), the ACC diagnosis was based on clinical presentation, mainly virilization, Cushing syndrome, and palpable abdominal mass. At diagnosis, 6 out of 33 patients (18%) had metastatic disease, including 2 ACC identified during cancer screening procedures [9,13]. Treatment data were available for 24 patients and follow-up data were available for 19. Two patients were not treated: one received only palliative care [18] and another died perinatally with a post-mortem diagnosis [19]. Seventeen patients underwent surgery alone, three of whom died: two due to ACC metastases [20,21] and one from yolk sac tumor [22]. Five patients received surgery, chemotherapy (CT), and mitotane; one of these patients died due to relapse 2 years later [14]. Wieneke score was calculated for 10 patients. In two ACC cases, the Wieneke score was ≤2, yet both demonstrated metastasis. One of these cases showed capsular and vascular invasion [9], that were not reported in the other case [23]. The details of all BWSp-ACC patients are reported in Table 1.

Five patients underwent molecular testing on tumor DNA: one exhibited a complex abnormal karyotype [21], one carried marked IC2-LoM [14], one showed a high level of genome-wide UPDpat [22], one exhibited LOH of chromosome 17p13 [24], and one carried a somatic pathogenic variant in the *CTNNB1* gene [13].

### 3.5. Comparison Between ACC and ACA/umACT Groups

**Figure 3:**
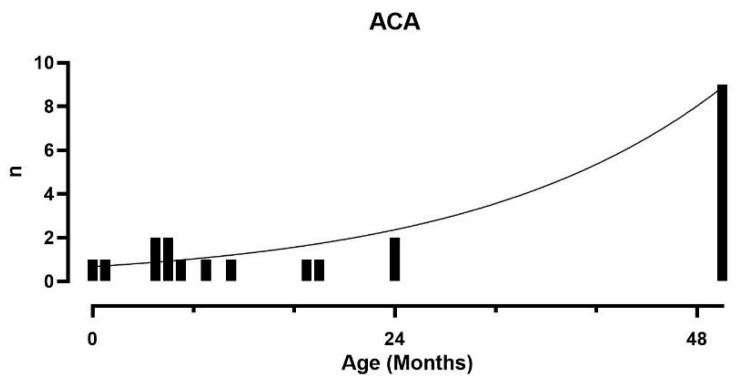
Distribution of age of diagnosis for ACA in BWSp patients.

In the comparison between the ACC and ACA/umACT groups, it was found that UPD(11)pat was significantly more frequent in the ACC population. Conversely, macroglossia and abdominal wall defects were more commonly observed in the ACA/umACT population. Additionally, ACAs were more frequently diagnosed through screening (Figure 3). No significant differences were found in the incidence of second tumors or overall mortality between the two groups. Follow-up data were available for 12 patients with ACA/umACT and 21 with ACC, with an average follow-up duration of 67.3 ± 66.3 months for ACA (range: 0–228 months) and 89.5 ± 106.4 months for ACC (range: 0–364 months). A follow-up duration of 0 months was considered only for deceased patients. Data are reported in Table 3.

**Table 1:** Details of 32 previously published and 1 unreported case of BWSp patients with ACC.

Reference	Year of Pubblication	Patient ID	Sex	Molecular Diagnosis	Wieneke Score	Age (years) at ACC Diagnosis	Localization	Method of ACC Diagnosis	Symptoms	Metastasis	Treatment	Last Follow-Up	BWSp Clinical Score	Years of Follow-Up	Associated Consitions
Pinto [13]	2021	case 5	F	UPD(11)pat	>4	4	Right	Symptoms	Abdominal pain	No	Surgery + CT + mitotane	Alive	2	17	
Weinstein [25]	1970		F	NA	NA	<1	Left	Symptoms	Palpable mass	No	Surgery	Alive	5	0	WT
Postema [22]	2019		F	GW-UPDpat	NA	37	Right	NA	NA	No	Surgery	Dead	4	2	Yolk sak tumor
Cöktü [26]	2020		F	UPD(11)pat	NA	<1	NA	NA	NA	NA	NA	Alive	NA	11	
Wijnen [14]	2012	case 1	F	IC2-LoM	NA	9	Left	Symptoms	Cushingoid features	No	Surgery + CT + mitotane	Dead	1	2	
Saypol [27]	1983		M	NA	NA	<1	Right	Symptoms	Cushingoid features	No	Surgery	Alive	4	30	
Sotelo-Avila [28]	1977		F	NA	NA	3.5	Bilateral	Symptoms	Cushingoid features	Yes	NA	NA	4	0	Adrenocortical cytomegaly
Fraumeni [29]	1967	case 2	M	NA	NA	<1	Right	Symptoms	Cushingoid features	NA	NA	Alive	5	1	
McDonnell [30]	2003	case 3	M	UPD(11)pat	NA	<1	Left	Symptoms	Cushingoid features	No	NA	NA	NA	0	
Eltan [31]	2020		F	IC2-LoM	NA	<1	Left	Symptoms	Cushingoid features	No	Surgery	Alive	2	1	
Pinto [13]	2021	case 3	F	UPD(11)pat	NA	<1	Left	Symptoms	Cushingoid features	No	Surgery	Alive	1	8	
Kostiainen [32]	2019	case 1	M	IC2-LoM	NA	<1	Right	Symptoms	Cushingoid features	NA	NA	Alive	2	0	
Benson [20]	1963	case 5	M	NA	NA	4	Right	Symptoms	Precocious Puberty	NA	Surgery	Dead	4	0	
Müller [33]	1978		F	NA	3	7.5	Right	Symptoms	Cushingoid features	No	Surgery	Alive	5	6.5	WT + Adrenocortical cytomegaly
Hertel [21]	2003		F	IC2-LoM	>4	4	Right	Symptoms	Cushingoid features	No	Surgery	Dead	3	8	
Pilloni [9]	2023		M	IC2-LoM	2	3	NA	Screening	Precocious Puberty	Yes (CNS)	Surgery + CT + mitotane	Alive	5	3	
Sherman [19]	1958		F	NA	NA	<1	Unilateral	Symptoms	Palpable mass	Yes (Lung, CNS)	Support	Dead	6	0	WT
Saracco [23]	1988		M	UPD(11)pat	≤2	0	Right	Symptoms	Cutaneous lesions	Yes (Skin, CNS)	Surgery	Alive	3	1	WT
Bertoin [24]	2015		F	GW-UPDpat	NA	16	Left	Symptoms	Virilization	No	Surgery	Alive	10	5	Adrenocortical cytomegaly
Sassi [34]	2021	case 2	M	UPD(11)pat	3	<1	Left	Symptoms	Abdominal syndrome	No	Surgery	Alive	4	0	
Federici [35]	1994	case 11	F	NA	NA	<1	Left	Symptoms	Virilization + abdominal mass	No	Surgery	Alive	1	2	
Pinto [13]	2021	case 4	F	UPD(11)pat	NA	1	Left	Symptoms	NA	Yes (Lung)	Surgery + CT + mitotane	Alive	1	27	
Pinto [13]	2021	case 2	M	UPD(11)pat	NA	2	Left	Screening	NA	Yes (Liver)	Surgery	Alive	3	11	
Miele [36]	2019	case 9	NA	UPD(11)pat	5	NA	NA	NA	NA	NA	Surgery + CT + mitotane	NA	4	0	
Henry [18]	1989		M	Dup11p15.5	NA	0	NA	NA	NA	NA	Support	Dead	NA	<1	
Gupta [37]	2018		NA	NA	NA	<1	Left	NA	NA	No	Surgery	Alive	NA	0	
Bliek [38]	2004		NA	UPD(11)pat	NA	NA	NA	NA	NA	NA	NA	NA	NA	0	
Brioude [39]	2013	case 10	F	UPD(11)pat	NA	<1	NA	NA	NA	NA	NA	NA	NA	0	
Brioude [39]	2013	case 22	F	UPD(11)pat	NA	3	NA	NA	NA	NA	NA	NA	NA	0	
Luca [40]	2015 2023		M	UPD(11)pat	NA	1	NA	NA	NA	NA	NA	Alive	4	19	
Present report	NA		F	UPD(11)pat	3	0	NA	Symptoms	Palpable mass	NA	Surgery	NA	9	0	
Kuznetsov [41]	2014		F	IC2-LoM	3	15	Bilateral	Symptoms	Virilization	No	Surgery	Alive	4	2	
Naotunna [42]	2023	case 3	M	NA	4	6	Right	Symptoms	Virilization	No	Surgery	NA	5	0	

Abbreviations: ACA—adrenocortical adenoma, ACT—adrenocortical tumor, Dup11p15.5—duplication of chromosome 11p15.5, IC2-LoM—imprinting center 2 loss of methylation, NA—not available, umACT—adrenocortical tumor of uncertain malignant potential, UPD(11)pat—paternal uniparental disomy of chromosome 11, WT—Wilms Tumor.

**Table 2:** Details of 22 previously published BWSp patients with ACA and umACT.

Reference	Year of Pubblication	Patient ID	Sex	Histology	Molecular Diagnosis	Wieneke Score	Age (years) at ACT Diagnosis	Localization	Method of ACT Diagnosis	Symptoms	Treatment	Last Follow-Up	BWSp Clinical Score	Years of Follow-Up	Associated Consitions
Cardinalli [43]	2012		F	ACA	NA	1	1.5	Monolateral	Screening	Cushingoid features	Surgery	Alive	8	0	myelolipoma
Doya [44]	2021		F	ACA	NA	2	1.5	Monolateral	Symptoms	Virilization	Surgery	Alive	4	2	
Schweiger [45]	2021		M	ACA	UPD(11)pat	1	<1	Monolateral	Symptoms	Cushingoid features	Surgery	Alive	NA	0	
Ben-Brahim [15]	2014		F	ACA	IC2-LoM	NA	<1	Monolateral	Symptoms	Cushingoid features	Surgery	Alive	NA	<1	
Pinto [13]	2021	case 1	F	ACA	UPD(11)pat	0	8.5	Bilateral	Symptoms	Virilization	Surgery	Alive	2	9.5	
Elnaw [46]	2019		F	ACA	NA	0	4	Monolateral	Symptoms	Virilization	Surgery	Alive	6	0	
Sbragia-Neto [47]	2000		M	ACA	NA	0	2	Monolateral	Symptoms	Virilization	Surgery	Alive	7	0	neonatal adrenal cysts
Clouston [48]	1989		F	ACA	NA	NA	45	Monolateral	Symptoms	Virilization	Surgery	Alive	6	3	
Schofield [16]	1995		M	ACA	Dup11p15.5	NA	<1	Monolateral	Symptoms	Palpable mass/cushing	Surgery	NA	6	0	
Wijnen [14]	2012	case 2	F	ACA	IC2-LoM	NA	14	Monolateral	Symptoms	Virilization	Surgery	Alive	0	0	
Alradadi [49]	2019		F	ACA	IC2-LoM	NA	<1	Bilateral	Symptoms	Virilization	Surgery	Alive	NA	8	
Mizota [50]	2005		F	ACA	NA	0	6	Bilateral	Symptoms	Virilization	Surgery	Alive	4	7	
Riedel [10]	1952		M	ACA	NA	NA	<1	Monolateral	Symptoms	Virilization	Surgery	Dead	5	6	WT
Gazzin [51]	2019	ID 21	M	ACA	IC2-LoM	NA	22	NA	Screening		Surgery	Alive	5	19	Sertoly tumor
Tlili-Graiess [11]	1994		F	ACA	NA	NA	<1	Monolateral	Screening		Surgery	Dead	8	0	
Vaughan [52]	1995		F	ACA	NA	NA	16	Monolateral	Screening		Surgery	Alive	NA	1.5	WT
Alsultan [53]	2008		F	ACA	IC2-LoM	NA	<1	Monolateral	Screening		Surgery	Alive	4	1.5	neuroblastoma
Giner [12]	2017		M	ACA	NA	NA	2	Ectopic	Screening		Surgery	Alive	NA	2	
Hayward [17] Byrne [54]	1988 1993	WtsAA	F	ACA	NA	NA	45	NA	NA		NA	NA	6	0	bicornate uterus + medullary sponge kidneys
Kim [55]	2019		M	umACT	UPD(11)pat	0	<1	Ectopic	Screening		Surgery	NA	5	0	
Jung [56]	2000		M	umACT	NA	4	0	Monolateral	Screening		Surgery	NA	6	0	
Pinto [13]	2021	case 6	F	umACT	UPD(11)pat	>4	11	Monolateral	Symptoms	Hypertension	Surgery	Alive	0	12	

Abbreviations: ACC—adrenocortical carcinoma, CT—chemotherapy, CNS—central nervous system, Dup11p15.5—duplication of chromosome 11p15.5, GW-UPDpat—genome-wide paternal uniparental disomy, IC2-LoM—imprinting center 2 loss of methylation, NA—not available, UPD(11)pat—paternal uniparental disomy of chromosome 11, WT—Wilms Tumor.

**Table 3:** Comparison of BWSp patients with ACA and umACT versus patients with ACC.

	ACA + umACT	ACC	*p*-Value
Overall mortality	2/13 (15.3%)	6/21 (28.6%)	NS
Mean age at diagnosis (years)	9.4	4	NS
Mean BWSp clinical score	4.8 ± 2.4	3.9 ± 2.1	NS
Molecular diagnosis			
IC2-LoM	5/10 (50%)	6/23 (26.1%)	NS
UPD(11)pat	4/10 (40%)	16/23 (69.6%)	NS
Method of ACT Diagnosis			
Symtomps	13/22 (59.1%)	22/33 (66.7%)	NS
Screening	8/22 (36.4%)	2/33 (6.1%)	0.010
BWSp typical features			
Macroglossia	12/17 (70.6%)	5/26 (19.2%)	0.0008
Abdominal wall defects	10/17 (58.8%)	5/26 (19.2%)	0.008
Lateralized overgrowth	15/17 (88.2%)	19/26 (73.1%)	NS
Malignancies (excluding ACC)	4/22 (18.2%)	3/33 (11.0%)	NS

Abbreviations: ACA—adrenocortical adenoma, ACT—adrenocortical tumor, IC2-LoM—imprinting center 2 loss of methylation, NS = non-significant, umACT—adrenocortical tumor of uncertain malignant potential, UPD(11)pat—paternal uniparental disomy of chromosome 11.

## 4. Discussion

Adrenal cortical tumors (ACTs) are rare in patients with BWSp when compared to other childhood neoplasms commonly associated with this syndrome, such as WT. While BWSp is well known for its association with multiple tumor types [1], the rarity of ACTs raises questions about the underlying mechanisms and the potential need for tailored surveillance strategies. In our literature review, we identified 33 BWSp patients with ACC, 19 with ACA, and 3 with umACT. These cases have been published in the literature over a long time span, i.e., from 1952 to 2023. Efforts were made to standardize the data as much as possible by calculating the Wieneke histopathological score [5] and the currently used BWSp clinical score [1] based on the information provided in the papers, with the intent of collecting the most extensive possible case series of BWSp patients affected by ACTs.

The BWSp clinical score was available for 26 patients with ACC and for 14 patients with ACA. The score was higher for patients with ACA (5.0 vs. 3.9), although this difference was not statistically significant, likely due to the small sample size (Table 3). However, it is worth noting that the diagnosis of ACC itself adds a point to the score, and 18 ACC patients had a score of ≤4, indicating that they did not meet the cutoff for clinical diagnosis before the onset of ACC. This finding supports previous observations [40,57] indicating that the currently used clinical score [1] is better at identifying patients with typical forms of BWSp, such as patients with IC2-LoM, who have a lower cancer risk. Conversely, it struggles to identify patients with atypical forms, who may have a higher cancer risk, such as some patients with UPD(11)pat. A comparison between the ACC and ACA populations revealed a statistically higher frequency of macroglossia and abdominal wall defects in ACA patients, consistent with the hypothesis that classical forms of BWSp are more prevalent in this population. Any future reassessment of the clinical criteria should take this into account and aim to implement strategies for identifying high-risk patients earlier in their clinical course.

The histopathological score currently used for ACTs, proposed by Wieneke and colleagues in 2003 [5], has suboptimal efficacy in pediatric ACTs [5,6]. We obtained a reliable Wieneke score for 10 ACC patients. Notably, two patients with a Wieneke score of ≤2, corresponding to benign histology, developed metastases [9,23]. One of these patients’ tumors exhibited vascular and capsular invasion (2 points in the Wieneke score), with a total score of 2 [9], indicating a possible greater significance of these features in predicting metastatic potential. However, neither capsular nor vascular invasion was present in the other patient [17]. Both of these patients with low histopathological scores and metastatic ACC exhibited an indolent course, with follow-ups (1 and 3 years) showing little to no progression of the metastases. Additionally, two other ACC patients who developed metastasis had, respectively, 11 and 27 years of follow-up free of disease progression [13]. Of the six metastatic ACC patients, follow-up data were available for five: one patient died shortly after birth with severe BWSp features and a post-mortem ACC diagnosis [19], while the other four exhibited medium to long follow-ups with an indolent course [9,13,23]. Two of these patients carried UPD(11)pat and one had IC2-LoM, while no molecular analysis was available for the fourth. Pediatric metastatic ACC has an overall estimated 5-year survival rate <20% [2], suggesting that these data are unusual and may indicate a tendency towards indolence in BWSp-related ACC. Nevertheless, given the limited follow-up data in terms of duration and patient numbers, these observations do not support definitive conclusions about tumor aggressivity and/or associated mortality.

Moreover, two BWSp-related ACC cases presented with central nervous system (CNS) metastasis in the absence of liver or lung involvement [11,23]: however, current guidelines do not recommend routine brain magnetic resonance imaging (MRI) for staging pediatric ACC in the absence of other metastatic sites or neurological symptoms [58]. Currently, brain MRI in such circumstances is only recommended for patients with Li–Fraumeni syndrome [58]. The evolution of these cases [11,23] suggests that such a recommendation should be extended to all pediatric ACC patients with a cancer predisposition syndrome, including BWSp. Additionally, the lack of correlation between the Wieneke score and malignancy in these two cases [11,23] raises questions about selecting ACA patients for comprehensive staging avoiding excessive medicalization. If on the one hand it seems excessive to perform a comprehensive staging on every child with an ACA, on the other hand, such an approach appears reasonable for patients with a cancer predisposition syndrome such as BWSp.

Current management guidelines for ACTs in BWSp patients, published in 2017 [59], recommend observation only with an ultrasound scan (USS) every 3 months for cystic masses at any age, as well as for solid masses identified within the first 6 months of life that show no suspicious features. Surgical treatment is recommended at any age for solid masses with suspicious features, as well as for any cases of solid masses after 6 months of age. This approach seems prudent for solid masses, for which surgical treatment is always recommended after 6 months of age. For cystic masses, only observation with USS every 3 months is suggested, since the majority of these cysts represent an adrenal cyst or hemorrhage. However, the case described by Pilloni and colleagues in 2023 [9], in which a cystic mass progressed to a metastatic solid ACC in less than the 3 months between USS, suggests a more cautious approach for cystic masses that do not undergo spontaneous regression in the early months of life. Overall, two metastatic ACC cases were diagnosed during USS cancer screening [9,13], suggesting that USS screening is not optimal for detecting ACC before metastasis and that surgery should be considered in all BWSp patients with cystic or solid persistent adrenal masses.

Overall, among the 12 BWSp patients with ACT who underwent molecular testing on tumor DNA, no common molecular pattern was identified. Three patients carried somatic pathogenic variants in the CTNNB1 gene [9,13], which have already been described in ACTs [2], while four patients exhibited marked IC2-LoM on tumor DNA [9,14,15]. The case presented by Pilloni in 2023 [9] demonstrated IC2-LoM in blood, with pronounced IC2 hypomethylation and additional IC1-GoM in the tumor, without UPD(11)pat (Figure 1), a finding comparable to cases reported in IC2-LoM BWSp patients with hepatoblastomas [57]. Given the role of IC1 in regulating IGF2, a growth factor critical in ACC pathogenesis [60], these molecular alterations highlight the importance of monitoring for malignancy in BWSp patients regardless of molecular subtype. In adult ACC, IGF2 overexpression linked to 11p15.5 abnormalities (e.g., loss of heterozygosity or UPD) is a common marker distinguishing ACC from ACA [60,61]; however, in pediatric ACTs, UPD(11)pat and IGF2 overexpression are found in both ACA and ACC [62,63]. These findings underscore the complex molecular landscape of ACTs in BWSp, with heterogeneity across patients and tumor types, suggesting that uniform surveillance strategies for all BWSp molecular subtypes are advisable, as recent guidelines suggest [64]. Considering ACC specifically, repeated DHEA-S measurement every 3 months, in conjunction with alpha-fetoprotein monitoring, may enhance early detection, as previously proposed [65].

This study has two main limitations. First, it employs a retrospective approach, incorporating data published over a 70-year time span. During this period, the diagnostic definitions and protocols for BWSp and ACTs have evolved significantly, resulting in variability in diagnostic approaches and follow-up data quality. This inherent variability presents challenges for direct data comparison and consistency in findings. Despite efforts to standardize data collection, this variability calls for prudent evaluation of the results, which would benefit from confirmation through additional observations. The second major limitation is the small sample size, which restricts the possibilities for statistical analysis and necessitates a descriptive approach. Nevertheless, this cohort represents the largest case series of BWSp patients with ACTs reported to date, given the rarity of both conditions.

## 5. Conclusions

In conclusion, we described 55 cases of ACTs in BWSp patients, highlighting the complexities of ACTs diagnosis and management in this population. Despite the limitations inherent in a retrospective design, the variability in diagnostic criteria and clinical management across decades, and a small sample size, our findings suggest that the current clinical BWSp scoring system may be inadequate for reliably identifying BWSp patients at higher risk for malignancy. Additionally, the existing histopathological scoring system for ACTs may not correlate consistently with malignancy in a subset of cases, confirming its known limitations in the pediatric setting. Furthermore, screening with USS does not appear adequate for the early diagnosis of ACC, and surgical intervention should always be considered. Finally, the results indicate a potential indolent course for ACCs in the BWSp population, challenging traditional prognostic expectations. These findings would benefit from confirmation in further studies aimed at elucidating the molecular mechanisms underlying ACTs in BWSp, with the goal of refining surveillance and management strategies for this at-risk patient population.

## Data Availability

The original contributions presented in the study are included in the article; further inquiries can be directed to the corresponding author.
